# Parental Evaluation of the Socio-Personal Adjustment of High Ability Students in a Cluster Grouping Program

**DOI:** 10.3390/children9010044

**Published:** 2022-01-02

**Authors:** María de los Dolores Valadez, Julián Betancourt, Triana Aguirre, Elena Rodríguez-Naveiras, África Borges

**Affiliations:** 1 Institute of Psychology and Special Education, Department of Applied Psychology, University Center for Health Sciences, University of Guadalajara, Guadalajara 44340, Mexico; dolores.valadez@academicos.udg.mx; 2 Jalisco Educational Center for High Abilities, Jalisco Education Secretary, Guadalajara 44100, Mexico; coordinacion@cepac.edu.jalisco.gob.mx; 3 Department of Clinical Psychology, Psychobiology and Methodology, University of La Laguna, 38200 San Cristóbal de La Laguna, Spain; naveiras@ull.edu.es (E.R.-N.); aborges@ull.edu.es (Á.B.)

**Keywords:** high ability, cluster grouping, parental evaluation

## Abstract

High ability students have differential cognitive characteristics that require a specific educational response to develop their full potential. Cluster ability grouping is one of the available approaches to respond to their training. One of the main criticisms of this teaching method, also supported by the big-fish-little-pond effect (BFLPE), is that high ability students may show a decrease in their self-concept. The aim of this research is to present the evaluation carried out by parents of primary school high ability students on the effect of an educational cluster grouping program on their personal and social adjustment, comparing these variables before the beginning of the school year and at the end of it. Approximately 100 students’ parents of the Educational Centre for Highly Ability Pupils participated in the evaluation. The instrument used for the evaluation was the Socialization Battery (BAS-2) for parents. The results showed that parents observed some improvements, either due to an increase in the scales measuring positive socialization variables or a decrease in the scales measuring negative socialization variables. The most substantial improvements are found in the second and third grades. We conclude that the results do not support the big-fish-little-pond effect (BFLPE).

## 1. Introduction

High ability students have differential cognitive characteristics that require a specific educational response to develop their full potential [1,2]. To support their educational needs, specific approaches have been adopted, such as in-school programs, which focus on the enrichment, acceleration, and cluster grouping, or after-school programs [3,4] and out-of-school [5].

Cluster grouping can be defined as an intervention for high ability or high achieving students which places together, in small groups or classrooms, a small number of students according to the initial assessments of their readiness, knowledge, interests, and abilities [6,7,8,9,10,11,12,13,14,15,16]. 

Many studies have investigated the effects of within-class ability grouping on the academic achievement of high ability students [17,18,19,20,21,22,23,24,25,26,27]. The findings show that the impact on academic achievement may or may not depend on the type of grouping implemented, the student’s socio-economic status, and their academic history [10,22,28].

According to [29], negative and non-significant results on academic performance in high ability children by using cluster grouping intervention might show that this approach on its own is not sufficient. What is more relevant is the so called “optimal match”, which appears when high ability students are offered a differentiated curriculum, full of challenges and in line with their interests and abilities, together with an educational environment that promotes their talents [18,30].

A more controversial subject is the effect that cluster grouping has on the personal and social adjustment of high ability students, an effect that has been the center of many investigations around their psychological and socioemotional well-being, focusing mainly on their self-concept and their self-esteem [6,8,24,30,31,32,33,34,35,36,37,38]. The results obtained by these investigations show different results. Some seem to find a very small positive impact on social self-concept among high-ability students participating in cluster grouping compared to those in a regular classroom. On the other side, whereas [39] consider that this type of clustering negatively affects self-concept, other researchers consider the decrease in self-concept because of clustering as being purely speculative [8,40,41,42].

Some researchers consider that cluster grouping can lead to a decrease in the self-concept of highly ability students and that this can be explained by the big-fish-little-pond effect (BFLPE) [8,24,35,39,43,44,45]. The BFLPE describes a frame of reference model affirming that self-perceptions in educational settings are largely determined by social comparison processes [4,46]. When highly ability students are subjected to a social comparison with a high ability reference group, such as students who also have high abilities, they experience a negative effect and their self-concept decreases [24,35,44,46].

Ref. [24] also consider that lower self-concept in high-ability students may be related to age, socio-economic status, or gender, among other factors. However, belonging to the high ability group may result in an increase their self-concept due to the so-called “basking in reflected glory effect” [38,41], which occurs when high ability students are in a classroom or school with students with similar characteristics. This can fill them with pride and with a sense of belonging; it can also make them strive for academic excellence, as well as strengthen their self-esteem and their school engagement [26,47,48,49].

Another research topic is how cluster grouping impacts students’ self-esteem; study [10] evaluated 13 meta-analysis studies describing the effects of cluster grouping on self-esteem in high ability students. The results showed a decrease in self-esteem scores. However, they were considered insignificant due to the low effect size. Study [10] also analyzed 11 studies of multilevel ability cluster grouping. The average effect size was very low or minimal for high ability students [10].

Then again, benefits in socioemotional aspects have also been reported, such as: (a) strong intrinsic motivation for learning; (b) a greater development of their interests and healthy social relationships [36,50,51]; (c) coexistence with peers with similar characteristics that strengthens their self-esteem and social skills; (d) opportunity to learn to work in groups and to configure a more accurate perception of their own socioemotional resources [41,52]; and (e) the problems of social isolation, peer rejection, loneliness and alienation that afflict extremely gifted students decrease with grouping as they feel accepted and equal to the rest of their group [25,53,54]. 

Therefore, it is essential to develop studies that analyze the effects of cluster grouping, with a rigorous evaluation. The evaluation of the psycho-pedagogical intervention for students with high abilities is a complex task that not only involves students and teachers but should also be carried out with parents’ participation, because their information can enrich the intervention [55], making them important agents for the evaluation of the changes.

Unfortunately, the number of studies on programs’ evaluation for parents of high ability children has been limited due to the lack of systematicity and research [56]. However, it is important to carry out these evaluations because it would help to understand whether these mentoring programs allow parents to identify and recognize the educational needs of their children, among other aspects, as well as to assess the effectiveness of the implemented programs [57].

In a study conducted with parents from three different countries, who attended programs for families of high ability children, they rated positively the content, the implementation time and the teachers who taught them, showing the relevance of the program. The authors conclude their work by pointing out the importance of promoting programs for parents as a strategy to provide greater support to their children [58].

Studies reviewing evaluation of programs for parents of high ability children highlight two important points: first, the need to implement these programs to respond to the demand of the parents to improve their knowledge about high abilities and to manage the different aspects of their children life, especially the academic, social, and emotional. Second, the importance of evaluating the program and its impact on parents, having reliable measurement instruments for this purpose [59,60].

The aim of this research is to present the evaluation made by parents of primary school students with high abilities on the effect of their personal and social adjustment, in an educational cluster grouping comparing these variables before the beginning of the school year and at the end of it. The contrasted hypothesis is that if any effect is shown, it will be positive, indicating improvements in personal and social adjustment at the end of the school year.

## 2. Method

### 2.1. Participants

The families (mothers and fathers) of 100 students of the Centre for Highly Ability Pupils (CEPAC) participated in the evaluation. The number of students in each grade was distributed as follows: 27 in first grade, 15 in second grade, 15 in third grade, 14 in fourth grade, 14 in fifth grade, and 15 in sixth grade. Each grade was initially composed of 15 students, except for first grade where there were two groups. The average age of the mother was 37.08 years (SD = 6.459 years) and the father’s mean age was 38.649 years (SD = 6.452). Table 1 shows the frequency of schooling and occupation of the parents of the 100 students of the Centre for Highly Ability Pupils who participated in the evaluation. 

### 2.2. Instruments

The Socialization Battery (BAS-2) [61] is a set of scales for parental assessment of children and adolescents’ socialization in school and out-of-school contexts. The items in the battery (114 in BAS-2) basically have two functions: (a) to obtain a socialization profile with seven scales, four of which related to positive-facilitating aspects (leadership, joviality, social sensitivity, and respect-self-control) and three with negative, disruptive, or inhibitory aspects (aggressiveness-stubbornness, apathy-withdrawal, and Anxiety-shyness); (b) to obtain an overall assessment of socialization with a socialization criterion scale. The socialization battery is appropriate for subjects aged 6 to 15 years old and can be completed by parents (BAS-2). The socialization battery is typed and allows us to obtain centiles for the scales of both versions, by sex and schooling. The higher the centile obtained in the facilitating scales, the greater the socialization. Conversely, the higher the centile in the disturbing scales, the greater the presence of aggressiveness-stubbornness, apathy-withdrawal and/or anxiety-shyness. Table 2 shows reliability results of the scale, which have been obtained by means of internal consistency and test-retest reliability.

### 2.3. Procedure

After the parents signed the informed consent form, they were asked to answer the Socialization Battery (BAS 2) at the beginning and end of the school year.

### 2.4. Data Analysis

Cronbach’s alpha was calculated to test the reliability of the scales in the sample. Student’s *t*-test for repeated measures was used to compare differences in students’ personal adjustment before and after the school year, using the statistical package for social sciences SPSS version 21.

## 3. Results

### 3.1. Instrument Reliability

The reliability of the instruments was calculated using Cronbach’s alpha. Table 3 presents the values obtained in the pre and posttest.

### 3.2. Comparison the Perception of Personal and Social Adjustment before and after the End of the School Year at CEPAC

To test the hypothesis that support the existence of a positive social impact on students with high abilities who entered CEPAC, we compared the results obtained in the pretest and posttest on the parents’ BAS-2 socialization questionnaire for each school grade. As we were dealing with seven variables to compare, Type I errors were corrected using Bonferroni’s correction (α = 0.05/7), producing a significance level of 0.007.

For the first grade of primary school, after applying the Bonferroni correction, no significant differences appear in any of the variables studied (see Table 4). However, in leadership and anxiety-shyness a small effect size was observed, which decreased at the end of the school year, whereas in joviality and respect-self-control the effect size, although also small, increased.

For the second grade, again no significant differences appear in any of the variables studied. However, the effect size is medium for joviality, social sensitivity, and respect-self-control and increase by the end of the school year. For aggressiveness-stubbornness and anxiety-shyness (see Table 5) the effect size is small and decline at the end of the school year.

In the third grade, the differences in the scales are again insignificant, but the effect size is medium in all them, except for the social sensitivity, where it is small (see Table 6). In all cases, the trend is in the same direction: scales indicating better fit are increasing and those indicating worse fit are decreasing.

For the fourth grade, significant differences were observed in joviality, where scores increased at the end of the school year. Significant differences were also observed for apathy-withdrawal, although in this case the scores decreased at the end of the school year and the effect size was large (see Table 7). A medium effect size is also observed for apathy-withdrawal and small effect sizes for social sensitivity and respect-self-control. 

Table 8 shows the results for the fifth grade. The differences were not significant in any of the any scales and whereas for leadership, social sensitivity, respect-self-control, aggressiveness-stubbornness and apathy-withdrawal the effect size was small, for joviality there was medium effect. 

For the sixth grade no significant differences were observed for any of the scales, although joviality, apathy-restraint and anxiety-shyness showed a medium effect size (Table 9).

## 4. Discussion

Regarding the hypothesis about the positive social impact on the high ability children who entered CEPAC, the results indicated that, according to the parents’ perception, at the end of the school year the students improved in facilitating aspects of socialization. The third, fourth, and fifth grade groups improved especially in joviality, whereas in the second grade group the improvement was in social sensitivity and respect-self-control. It is also important to note that the disruptive aspects decreased, particularly in the second, third, and fourth grade groups, which shows that clustering facilitates socialization because students live together with others like them. Even first grade students decreased their leadership, probably because they saw themselves among peers.

Although one of the main concerns of parents is the education of their children, they are also concerned about their socialization and adjustment [59,60]. Although it has been shown that high ability students as a group do not present difficulties in this area, attending a grouped school allowed for increasing the facilitating aspects of socialization and decreasing the disruptive ones that were present previously.

The main finding of this study is the absence of negative effects on students participating in a cluster grouping program. This is a relevant result, as it contradicts [38] results and supports studies pointing to the absence of negative effects [8,40,41,42]. However, it may help to clarify the growing debate the specialized literature on the adverse effects produced by this type of program.

These results are encouraging. Education with students with similar intellectual ability not only has no disruptive effects but also appears to be a very important socialization agent.

The principal limitation of this study is that only one educational center was analyzed without a control group that would allow us to understand whether there are differences in these variables when high ability students are educated in classrooms with peers of different intellectual abilities.

Moreover, given that CEPAC receives students every year, not only from elementary school, but also from middle and even high school, it is very important to continue these evaluations, in order to verify the effects of the grouping on the personal and social adaptation of the students with longitudinal and cross-sectional analysis.

## 5. Conclusions

In conclusion, parents perceive that their children’s socialization increase after studying in a cluster grouping for one year. 

The increase is due to the school’s encouragement of the development of socioemotional skills in students and the creation of future active citizens, concerned with ethical and social problems of the world in which they live. 

It is also evident that grouping not only has an impact at the academic level but also at the social level, particularly in aspects related to leadership, joviality, respect, self-control, among others.

## Figures and Tables

**Table 1:** Fathers’ and mothers’ schooling and type of employment.

Schooling	Fathers	Mothers	Type of Employment	Fathers	Mothers
Primary education	14	8	Labourer (or workman)	3	-----
High school	19	22	Employee	36	24
Graduate	55	56	Professional	33	36
Postgraduate	9	11	Businessman	12	5
No data	3	3	Technician	4	------
			Household	------	35
			No data	12	------
Total	100	100	Total	100	100

**Table 2:** Reliability presented in the BAS-2 manual.

BAS-2	Le	Jo	Ss	Rsc	Ast	Aw	As	Le
Internal Consistency	83	80	82	86	84	83	82	79
Test-retest	58	55	54	61	67	70	66	69

Note: Le = Leadership; Jo = Joviality; Ss = Social Sensitivity; Rsc = Respect-Self-Control; Ast = Aggressiveness-Stubbornness; Aw = Apathy-Withdrawal; As = Anxiety-Shyness.

**Table 3:** Reliability of the BAS-2 scales.

Aplication	Le	Jo	Ss	Rsc	Ast	Aw	As
Pre-Test	0.87	0.90	0.88	0.91	0.85	0.82	0.81
Post-Test	0.84	0.91	0.87	0.92	0.84	0.85	0.82

Note: Le = Leadership; Jo = Joviality; Ss = Social Sensitivity; Rsc = Respect-Self-Control; Ast = Aggressiveness-Stubbornness; Aw = Apathy-Withdrawal; As = Anxiety-Shyness.

**Table 4:** Scores obtained in the parents’ BAS at the beginning and end of the first grade of primary school.

Scales	Pre-Test		Post-Test					
	Mean	SD	Mean	SD	*t*	gl	*p*	d
Le	41.22	5.720	39.00	8.981	2.356	26	0.026	0.453
Jo	30.59	3.661	31.74	4.399	−1.312	26	0.201	−0.252
Ss	27.48	5.720	25.56	6.123	−0.053	26	0.958	−0.010
Rsc	38.11	7.454	38.89	8.149	−1.151	26	0.260	−0.221
Ast	7.59	5.976	7.04	5.481	0.500	26	0.621	0.096
Aw	3.48	3.446	3.78	4.644	−0.423	26	0.675	−0.081
As	6.07	3.362	5.33	3.063	1.243	26	0.225	0.239

Note: Le = Leadership; Jo = Joviality; Ss = Social Sensitivity; Rsc = Respect-Self-Control; Ast = Aggressiveness-Stubbornness; Aw = Apathy-Withdrawal; As = Anxiety-Shyness.

**Table 5:** Scores obtained in the parents’ BAS at the beginning and end of the second grade of primary school.

Scales	Pre-Test		Post-Test					
	Mean	SD	Mean	SD	*t*	gl	*p*	d
Le	41.87	6.379	42.40	6.266	−0.431	14	0.673	−0.111
Jo	31.33	4.483	33.20	3.385	−1.943	14	0.072	−0.501
Ss	28.87	6.368	32.33	7.961	−2.606	14	0.021	−0.672
Rsc	36.13	8.123	41.07	9.453	−2.486	14	0.026	−0.641
Ast	8.60	7.099	5.43	4.627	2.486	14	0.026	0.641
Aw	2.13	1.995	2.07	3.390	0.076	14	0.941	0.019
As	6.33	3.619	4.87	2.475	1.682	14	0.115	0.434

Note: Le = Leadership; Jo = Joviality; Ss = Social Sensitivity; Rsc = Respect-Self-Control; Ast = Aggressiveness-Stubbornness; Aw = Apathy-Withdrawal; As = Anxiety-Shyness.

**Table 6:** Scores obtained in the parents’ BAS at the beginning and end of the third grade of primary education.

Scales	Pre-Test		Post-Test					
	Mean	SD	Mean	SD	*t*	gl	*p*	d
Le	39.00	8.544	36.00	7.151	2.254	14	0.041	0.582
Jo	27.27	7.116	30.20	3.821	−2.155	14	0.049	−0.557
Ss	29.27	7.450	28.13	6.243	1.139	14	0.274	0.294
Rsc	37.27	10.173	41.40	8.131	−2.199	14	0.045	−0.568
Ast	4.73	6.703	5.93	7.126	2.901	14	0.012	0.749
Aw	8.00	7.865	3.33	4.850	2.606	14	0.021	0.673
As	8.67	6.388	4.87	4.868	2.816	14	0.014	0.727

Note: Le = Leadership; Jo = Joviality; Ss = Social Sensitivity; Rsc = Respect-Self-Control; Ast = Aggressiveness-Stubbornness; Aw = Apathy-Withdrawal; As = Anxiety-Shyness.

**Table 7:** Scores obtained in the parents’ BAS at the beginning and end of the fourth grade of primary school.

Scales	Pre-Test		Post-Test					
	Mean	SD	Mean	SD	*t*	gl	*p*	d
Le	37.57	7.251	36.50	7.251	0.627	13	0.541	0.168
Jo	25.36	7.023	29.43	4.815	−3.818	13	0.002	−1.020
Ss	27.00	7.200	28.21	6.507	−0.764	13	0.459	−0.204
Rsc	36.36	9.337	38.71	5.837	−1.074	13	0.302	0.287
Ast	9.71	8.624	8.36	7.196	0.673	13	0.512	0.180
Aw	10.07	6.855	3.64	2.818	3.313	13	0.006	0.885
As	7.79	4.726	5.64	2.437	2.206	13	0.046	0.590

Note: Le = Leadership; Jo = Joviality; Ss = Social Sensitivity; Rsc = Respect-Self-Control; Ast = Aggressiveness-Stubbornness; Aw = Apathy-Withdrawal; As = Anxiety-Shyness.

**Table 8:** Scores obtained in the parents’ BAS at the beginning and end of the fifth grade of primary school.

Scales	Pre-Test		Post-Test					
	Mean	SD	Mean	SD	*t*	gl	*p*	d
Le	42.00	6.139	40.29	5.622	1.008	13	0.332	0.269
Jo	29.93	5.717	33.14	3.900	−2.330	13	0.037	−0.622
Ss	30.07	6.183	31.43	6.813	−0.919	13	0.375	−0.245
Rsc	38.29	7.322	42.57	7.663	−1.785	13	0.098	−0.477
Ast	6.50	3.838	8.57	8.401	−0.958	13	0.356	−0.256
Aw	2.93	1.730	4.43	3.817	−1.623	13	0.129	−0.433
As	5.36	4.343	5.71	3.771	−0.354	13	0.729	−0.094

Note: Le = Leadership; Jo = Joviality; Ss = Social Sensitivity; Rsc = Respect-Self-Control; Ast = Aggressiveness-Stubbornness; Aw = Apathy-Withdrawal; As = Anxiety-Shyness.

**Table 9:** Scores obtained in the parents’ BAS at the beginning and end of sixth grade of primary school.

Scales	Pre-Test		Post-Test					
	Mean	SD	Mean	SD	*t*	gl	*p*	d
Le	40.53	6.685	39.27	5.496	0.659	14	0.521	0.170
Jo	27.40	7.109	31.40	5.565	−2.131	14	0.051	−0.550
Ss	31.87	10.398	32.53	7.110	−0.232	14	0.820	−0.059
Rc	42.60	8.467	42.67	5.367	−0.035	14	0.973	−0.008
Ast	5.27	4.728	7.87	9.448	−0.898	14	0.384	−0.231
Aw	5.73	5.431	3.20	3.764	1.792	14	0.095	0.462
As	6.60	3.521	5.13	1.846	1.361	14	0.195	0.351

Note: Le = Leadership; Jo = Joviality; Ss = Social Sensitivity; Rsc = Respect-Self-Control; Ast = Aggressiveness-Stubbornness; Aw = Apathy-Withdrawal; As = Anxiety-Shyness.

## Data Availability

The data presented in this study are available upon request from the corresponding author, due to restrictions, privacy, and ethical constraints.

## References

[1] Rodríguez-Naveiras E., Cadenas M., Borges Á., Valadez D. (2019). Educational Responses to Students with High Abilities from the Parental Perspective. Front. Psychol..

[2] Sastre-Riba S., Fonseca-Pedrero E., Santarén-Rosell M., Urraca-Martínez M. (2015). Evaluation of satisfaction in an extracurricular enrichment program for high-intellectual ability participants. Psicothema.

[3] Kelemen G. (2012). Identification of highly gifted children. Exedra.

[4] Sánchez E. (2011). Los Niños Superdotados: Una Aproximación a su Realidad.

[5] Rodríguez-Naveiras E., Díaz M., Rodríguez M., Borges A., Valadez M.D. (2015). Programa Integral Para Altas Capacidades. Descubriéndonos: Una Guía Para su Aplicación.

[6] Beker M., Neumann M., Tetzner J., Böse S., Knoppick H., Maaz K., Baumert J., Lehmann R. (2014). Is Early Ability Grouping Good for High-Achieving Students’ Psychosocial Development? Effects of the Transition into Academically Selective Schools. J. Educ. Psychol..

[7] Gentry M. (1996). Cluster Grouping: An Investigation of Student Achievement, Identification, and Classroom Practice. Doctoral Thesis.

[8] Gross M.U.M. (2004). Exceptionally Gifted Children.

[9] Gross M.U.M., Smith S.R., Smith S.R. (2021). Put Them Together and See How They Learn! Ability Grouping and Acceleration Effects on the Self-Esteem of Academically Gifted High School Students. Handbook of Giftedness and Talent Development in the Asia-Pacific.

[10] Kulik J.A., Kulik C.L.C. (1992). Meta-analytic findings on grouping programs. Gift. Child Q..

[11] Lleras C., Rangel R. (2009). Ability Grouping Practices in Elementary School and African American/Hispanic Achievement. Am. J. Educ..

[12] Marsh H.W., Craven R.G. (2006). Reciprocal effects of self-concept and performance from a multidimensional perspective: Beyond seductive pleasure and unidimensional perspectives. Perspect. Psychol. Sci..

[13] Missett T.C., Brunner M.M., Callahan C.M., Moon T.R., Azano A.P. (2014). Exploring teacher beliefs and use of acceleration, ability grouping, and formative assessment. J. Educ. Gift..

[14] Stanley J.C. (1991). A better model for residential high schools for talented youths. Phi Delta Kappan.

[15] Stanley J.C., Benbow C.P. (1982). Educating Mathematical y Precocious Youths: Twelve Policy Recommendations. Educ. Res..

[16] Sternberg R. (2020). Transformational Giftedness: Rethinking Our Paradigm for Gifted Education. Roeper Rev..

[17] Adelson J.L., Brittany D.C. (2011). Grouping for achievement gains: For whom does achievement grouping increase kindergarten reading growth?. Gift. Child Q..

[18] Bolick M., Rogowsky B. (2016). Ability Grouping is on the Rise, but Should It Be. J. Educ. Hum. Dev..

[19] Brulles D., Peters S.J., Saunders R. (2012). School-wide mathematics achievement within the gifted cluster grouping model. J. Adv. Acad..

[20] Kulik J.A., Kulik C.L.C. (1987). Effects of ability grouping on student achievement. Equity Excell..

[21] Kulik J.A., Kulik C.L.C., Colangelo N., Davis G.A. (1991). Grouping capacity and gifted students. Handbook of Education for the Gifted.

[22] Leonard J. (2001). How group composition influenced the achievement of sixth-grade mathematics students. Math. Think. Learn..

[23] Nomi T. (2010). The effects of within-class ability grouping on academic achievement in early elementary years. J. Res. Educ. Eff..

[24] Preckel F., Schmidt I., Stumpf E., Motschenbacher M., Vogl K., Vsevolod S., Schneider W. (2019). High-Ability Grouping: Benefits for Gifted Students’ Achievement Development without Costs in Academic Self-Concept. Child Dev..

[25] Steenbergen-Hu S., Matthew C., Olszewski-Kubilius P. (2016). Summary: What One Hundred Years of Research Says about the Effects of Ability Grouping and Acceleration on K-12 Students’ Academic Achievement. Acad. Res..

[26] Trautwein U., Ludtke O., Baumert J. (2006). Self-Esteem, Academic Self-Concept, and Achievement: How the Learning Environment Moderates the Dynamics of Self-Concept. J. Personal. Soc. Psychol..

[27] Winebrenner S., Brulles D. (2008). Cluster Grouping Handbook: Using Cluster Grouping to Challenge Gifted students and Improve Schoolwide Achievement.

[28] Gentry M., Owen S.V. (1999). An investigation of total school flexible cluster grouping on identification, achievement, and classroom practices. Gift. Child Q..

[29] Herderson H. (2007). Multi-level selective classes for gifted students. Int. Educ. J..

[30] Sternberg R.J., Ambrose D. (2021). Conceptions of Giftedness and Talent.

[31] Behrend A. (2012). Self-Perceptions of Gifted Achievers and Achievers and Underachievers: A Phenomenological Study. Doctoral Dissertation.

[32] Castle S., Deniz C.B., Tortora M. (2005). Flexible grouping and student learning in a high-needs school. Educ. Urban Soc..

[33] Delcourt M.A.B., Cornell D.G., Goldbert M.C. (2007). Cognitive and affective 139 learning outcomes of gifted elementary school students. Gift. Child Q..

[34] Gentry M. (2014). Total, School Cluster Grouping: A Comprehensive, Research-Based Plan for Raising Student achievement and Improving Teacher Practices.

[35] Marsh H.W., Parker J.W. (1984). Determinants of self-concept: Is it better to be a relatively large fish in a small pond even if you don’t learn to swim as well?. J. Pers. Soc. Psychol..

[36] Neihart M. (2007). The Socioaffective Impact of Acceleration and Ability Grouping: Recommendations for Best Practice. Gift. Child Q..

[37] Preckel F., Niepel C., Schneider M., Brunner M. (2013). Self-concept in adolescence: A longitudinal study on reciprocal effects of self-perceptions in academic and social domains. J. Adolesc..

[38] Seaton M., Marsh H.W., Craven R.G. (2009). Earning its place as a pan-human theory: Universality of the big-fish-little-pond effect across 41 culturally and economically diverse countries. J. Educ. Psychol..

[39] Marsh H.W., Hau K.T. (2003). The big-fish effect on academic self-concept: A cross-cultural (26-country) test of the negative effects of academically selective schools. Am. Psychol..

[40] Dai Y.D., Rinn A.N. (2008). The big-fish-little-pond effect: What do we know and where do we go from here?. Rev. Educ. Psychol..

[41] Gentry M. (2016). Commentary on “Does sorting students improve scores? An analysis of class composition”. J. Adv. Acad..

[42] Henshon S. (2020). Leading the way toward the future: An interview with marcia gentry. Roeper Rev..

[43] Herman J., Schminn I., Kessels U., Preckel F. (2016). Big fish in big ponds: Contrast and assimilation effects on mathand verbalself-concepts of students in within-school gifted tracks. Br. J. Educ. Psychol..

[44] Preckel F., Brull M. (2008). Grouping the gifted and talented: Are gifted girls more likely to suffer The consequences?. J. Educ. Gift..

[45] Preckel F., Brull M. (2010). The benefit of being a big fish in a big pond: Contrast and assimilation effects on academic self-concept. Learn. Individ. Differ..

[46] Huguet P., Dumas F., Marsh H. (2009). Clarifying the role of social comparison in the big-fish-little-pond effect (bflpe): An integrative study. J. Personal. Soc. Psychol..

[47] Hattie J.C. (2002). Classroom composition and peer effects. Int. J. Educ. Res..

[48] Hattie J.C. (2009). Visible Learning: A Synthesis of over 800 Meta-Analyses Relating to Achievement.

[49] Ridgley L.M., Rubenstein L., Callan G.L. (2020). Gifted underachievement within a self-regulated learning framework: Proposing a task-dependent model to guide early identification and intervention. Psychol. Sch..

[50] Adams-Byers J., Whitsell S.S., Moon S.M. (2004). The perceptions of students endowed with the academic and social/emotional effects of homogeneous and heterogeneous grouping. Yrly. Gift. Child.

[51] Kuriloff P., Reichert M.C. (2003). Boys of class. Boys of Color: Negotiating the academic and social geography of an elite independent school. J. Soc. Issues.

[52] Gentry M., Callahan C.M., Hertzberg-Davis H.L. (2018). Cluster grouping. Fundamentals of Gifted Education: Considering Multiple Perspectives.

[53] Goldsmith P.R. (2011). Coleman revisited: School segregation, companions, and frog ponds. Am. Educ. Res. J..

[54] Silverman L.K. (1993). Counseling families. Counseling the Gifted and Talented.

[55] Pfeiffer S.I. (2015). Essentials of Gifted Assessment.

[56] Leana-Tascilar M.Z., Ozyaprak M., Yilmaz O. (2016). An online training program for gifted children’s parents in Turkey. Eurasian J. Educ. Res..

[57] Costa-Lobo C., Pinho-Pereira S., Vestena C., Piske F.H.R., Stoltz T., Vázquéz-Justo E. Impact of a psychological intervention with parents of gifted students. Proceedings of the INTED2019 Conference.

[58] Rodríguez-Naveiras E., Borges A. (2020). Programas extraescolares: Una alternativa a la respuesta educativa de altas capacidades. Rev. Educ. Desarro..

[59] Flores-Bravo J.F., Valadez M.D., Borges A., Betancourt J. (2018). Principales preocupaciones de padres de hijos con altas capacidades. Rev. Educ. Desarro..

[60] Valadez M.D., Betancourt J., Ortiz G., Flores J.F., Almanza C. (2019). Preocupaciones de padres de hijos adolescentes con altas capacidades. Sobredotação.

[61] Silva F., Martorell M. (1989). BAS 1-2 Batería de Socialización (Para Profesores y Padres).

